# Epidemiology, Associated Factors and Implications for Effective Control of Pediculosis Among Primary Schoolgirls in Thailand: A Cross-Sectional Study

**DOI:** 10.3390/insects17040413

**Published:** 2026-04-10

**Authors:** Manachai Yingklang, Patchana Hengboriboonpong Jaidee, Penchom Janwan, Wanchai Maleewong, Na T. D. Tran, Tongjit Thanchomnang

**Affiliations:** 1 Faculty of Public Health, Burapha University, Chonburi 20131, Thailand; manachai.yi@buu.ac.th (M.Y.); patchana@buu.ac.th (P.H.J.); 2 Department of Medical Technology, School of Allied Health Sciences, Walailak University, Nakhon Si Thammarat 80160, Thailand; pairwu@gmail.com; 3 Department of Parasitology, Faculty of Medicine, Khon Kaen University, Khon Kaen 40002, Thailand; wanch_ma@kku.ac.th; 4 Mekong Health Science Research Institute, Khon Kaen University, Khon Kaen 40002, Thailand; 5 Faculty of Medical Laboratory Science, Danang University of Medical Technology and Pharmacy, Danang 550000, Vietnam; ttdna@dhktyduocdn.edu.vn; 6 Biomedical Science Research Unit, Faculty of Medicine, Mahasarakham University, Maha Sarakham 44000, Thailand

**Keywords:** head lice, risk factor, child health, public health, epidemiology, pediculosis

## Abstract

Pediculosis remains a public health problem among primary schoolchildren worldwide, including in Thailand. This study aimed to determine the prevalence of pediculosis and identify associated determinants among primary schoolgirls from different socio-geographic regions of Thailand to inform effective control strategies. A cross-sectional survey was conducted among 494 schoolgirls from eastern, northeastern, and southern provinces. Data on demographic characteristics, socioeconomic status, personal hygiene practices, parental knowledge and attitudes toward head lice, and school health policies were collected using questionnaires and interviews with school administrators. Univariable analyses and a generalized linear mixed model (GLMM) with school as a random effect were used to account for clustering. The overall prevalence of pediculosis was 50.81% (95% CI: 46.31–55.20), with significant variation across provinces. In univariable analysis, several factors were associated with infestation. However, after accounting for clustering, only class level (adjusted OR = 3.09; 95% CI: 1.31–7.29) and self-performed hair washing (adjusted OR = 2.93; 95% CI: 1.57–5.49) remained significantly associated with pediculosis, while other associations were attenuated. Parental knowledge was moderate, and commonly held beliefs regarding prevention and treatment varied. None of the participating schools had routine head lice screening policies. These findings indicate that pediculosis is likely influenced by both individual and school-level factors. Control efforts may benefit from coordinated school-based approaches, alongside improved access to effective treatment and targeted health education.

## Simple Summary

Pediculosis (head lice infestation) remains a persistent public health problem among primary schoolchildren in Thailand, particularly among schoolgirls. This study assessed the prevalence of pediculosis and explored associated factors across different socio-geographic regions and informed control strategies. A cross-sectional survey of 494 primary schoolgirls from eastern, northeastern, and southern provinces was conducted. Information on infestation status, demographic characteristics, socioeconomic factors, hygiene practices, parental beliefs, and school contexts was collected. The overall prevalence of pediculosis was high (50.81%) and varied across study areas. Univariable analyses suggested that several factors, including hair-washing practices, household income, and use of herbal treatments, were associated with pediculosis. However, after accounting for clustering at the school level, only class level and self-performed hair washing remained consistently associated, while other associations were attenuated. This suggests that some observed differences may reflect contextual factors within schools rather than individual behaviors alone. Parents had a fair amount of knowledge, but their ideas about prevention and treatment were different and not well-supported by evidence. None of the participating schools had routine head lice screening programs. These findings suggest that pediculosis may be influenced by both individual-level and school-level factors and that control strategies should extend beyond personal hygiene to include coordinated, school-based interventions tailored to local contexts.

## 1. Introduction

The head louse (*Pediculus humanus capitis*) is an obligate ectoparasite that feeds on human blood and causes pediculosis. The life cycle of head lice consists of three stages: egg, nymph, and adult. Adult lice can persist on human hosts for up to 30 days [1]. Although they cannot jump or fly, head lice can spread through direct head-to-head contact or indirectly through infested objects such as hairbrushes, clothing, hats, towels, or combs [2]. Head louse infestations can occur in individuals of all ages and hair types. However, they are more commonly observed in children, particularly in school and home settings, where they can become a recurring problem if not properly addressed [3,4]. Globally, it is estimated that head lice infest approximately 19% (95% CI: 18.0–20.0) of schoolchildren, with higher rates reported among girls aged 3 to 12 years old [5,6]. Low-intensity infestations are generally less troublesome, and affected individuals may tolerate them to some extent, while chronic infestations can result in severe consequences, such as scalp scaling and secondary bacterial infections from scratching [7,8,9,10].

The control and management of head lice, including the use of pediculicidal treatments and manual removal, are complex processes that require research evidence on local epidemiology [11,12]. Manual removal is time-consuming, while pediculicide treatments often need to be repeated 7 to 10 days later to eliminate newly hatched nymphs [13]. Moreover, head lice can survive under adverse conditions, including water immersion, for several hours [14,15,16]. The development of resistance to many pediculicide treatments has further complicated the management of head lice [17,18,19,20]. Understanding the relative risk factors and the distribution of susceptible and/or resistant lice is crucial for the development of effective control strategies.

Various factors associated with pediculosis is well known. These factors encompass personal characteristics of children, such as hair styles, educational levels, age, and gender [21]. Behaviors, including frequency of hair washing and engagement in playing activities, have also been reported to influence the risk of infestations, as do living conditions (such as inadequate access to water or bathrooms) [22,23,24,25,26]. In developed countries with good hygiene and high income, the prevalence of pediculosis is much lower than in developing countries or poor communities [27,28]. However, the relationship between increasing living standards and a decrease in head louse prevalence remains controversial.

In Thailand, head lice management commonly relies on over-the-counter pediculicides, particularly permethrin (0.5–1%) and carbaryl (0.6%), which are widely available. In addition, herbal-based treatments derived from local plants, such as *Annona squamosa*, are frequently used. In routine practice, treatments are typically administered by caregivers and may vary in terms of application technique, duration, and adherence to retreatment recommendations. Furthermore, many commonly used products have limited ovicidal activity, and reduced susceptibility to pyrethroid-based pediculicides has been increasingly reported [29,30]. These factors may contribute to incomplete eradication and recurrent infestation in real-world settings.

Despite Thailand’s status as a developing country with improving access to healthcare and washing facilities, the prevalence of pediculosis remains high throughout the country, especially among girl schoolchildren. In 1988, rural areas in the northeastern part of Thailand exhibited the highest prevalence of pediculosis (57.7%), followed by the southern (52.3%), central (44.1%), and northern (36.6%) regions [31]. Currently, regional prevalence rates range widely, from 15.1% to 86.1%, with some controversy about factors influencing prevalence [32,33,34,35]. These factors may include personal characteristics, hygiene practices, social environment, economic, and healthcare factors [36]. However, studies on pediculosis in Thailand have yet to clarify the importance of specific factors and resolve inconsistent findings.

In this study, we aimed to investigate the prevalence of pediculosis and factors associated with this among girl schoolchildren from different socio-geographic backgrounds of Thailand. Although pediculosis is not a gender-specific disease, infestation among boys in Thai primary schools is uncommon due to mandatory short-hair regulations, which substantially reduce exposure and transmission risk. This study intentionally focused on schoolgirls, who represent the highest-risk group, to provide more informative estimates of prevalence and associated factors. Additionally, we sought to assess parental knowledge and attitudes towards head louse infestations, as well as the existing health policies governing screening and treatment programs for head lice in schools. Understanding the underlying determinants of health associated with pediculosis is crucial for developing targeted interventions and policies to effectively reduce the burden of this condition.

## 2. Materials and Methods

### 2.1. Study Design, Population, and Eligibility Criteria

A cross-sectional study was conducted between January and March 2023, involving eight public primary schools located in three different regions of Thailand: eastern (Chonburi Province), northeastern (Maha Sarakham Province), and southern (Nakhon Si Thammarat Province). These provinces were chosen based on their differing social and geographic characteristics. Chonburi Province is renowned for its industrial and tourism sectors. It is one of the three provinces comprising the Eastern Economic Corridor of Thailand, which fosters extensive business development. Maha Sarakham Province, located in the northeastern region, has an economy that relies heavily on agricultural activities, especially rice farming. Nakhon Si Thammarat Province, in southern Thailand, is situated on the Malay Peninsula. The province has a tropical climate characterized by two distinct seasons—wet and dry. The economy relies on agriculture, industry, and mining. Most inhabitants of this province are Thai, followed by Thai-Muslim.

A multi-stage sampling approach was applied to select study schools (File S1). In the first stage, three provinces were purposively selected to represent different geographic regions and socio-contextual settings: Chonburi Province (eastern region), Maha Sarakham Province (northeastern region), and Nakhon Si Thammarat Province (southern region). In the second stage, one district within each province was selected using simple random sampling. A complete list of all public primary schools in each selected district was obtained from the respective provincial education office and used as the sampling frame. Schools were screened based on predefined eligibility criteria, including being government-administered institutions offering primary-level education, having at least 50 girl students, and providing institutional agreement to participate during the study period and data collection procedures. All eligible schools were enumerated, and a subset was selected using a simple random sampling procedure. In the third stage, a total of eight schools were included across the three provinces (Chonburi: 2; Nakhon Si Thammarat: 3; Maha Sarakham: 3). Schools in Nakhon Si Thammarat and Maha Sarakham were located in rural areas, whereas those in Chonburi were located in urban areas. In the final stage, all eligible schoolgirls aged 3–12 years within the selected schools were invited to participate.

The study population was limited to girls in primary school, guided by previous evidence suggesting a higher prevalence of pediculosis among girls than boys in Thailand [32,33,34,35]. Infestation among boys has been reported to be rare or negligible in many settings, which is commonly attributed to shorter hair length (crew-cut hairstyles) and reduced head-to-head contact. Therefore, focusing on girls allowed for a more efficient assessment of risk factors within the population most affected. Therefore, restricting the study population to girls was a predefined methodological decision.

### 2.2. Sample-Size Calculation

The sample size was calculated based on the prevalence (p) of pediculosis in Thailand in 2018 of 58.60% [37], with a 95% confidence interval (z = 1.96) and margin of error (d = 0.05). The calculated sample size was 375 individuals. A total of eight school directors and 510 girl children (aged 3–12 years), as well as their parents/guardians, were willing to participate in this study and were included.

### 2.3. Detection of Head Lice

After obtaining written informed consent forms and completion of the questionnaire, a plastic fine-tooth comb was used to comb through the hair of all children at the school, starting from the scalp and moving toward the ends of the hair. This procedure was applied consistently across all study sites to ensure standardized detection. Screening was conducted in private settings. Parents were informed of the measures in place to protect their children’s privacy and dignity. Trained staff carefully combed live lice onto a sheet of white paper. Active infestation was defined as the presence of at least one live louse (nymph or adult). Head louse eggs were identified either with the naked eye or with the assistance of a magnifying glass. Live eggs were white or yellow in color, possessing an intact operculum, and were found attached to the hair shaft less than 1 cm from the scalp, particularly behind the ears and at the back of the neck. If only eggs were found and they were located more than 1 cm away from the scalp, it was considered a negative infestation [38]. At the end of this study, all infested children were treated with 1% permethrin and/or 0.6% carbaryl shampoos as appropriate. Educational sessions were held in schools to normalize pediculosis as a common condition and reduce stigma.

### 2.4. Data Collection and Measurement

The demographic characteristics of schoolgirl children, including their class level and hairstyle, were recorded, along with their head louse infestation status and personal hygiene practices (performed by themselves or parents/guardians). The questionnaire administered to the parents/guardians collected information on their demographic characteristics (age, marital status, and gender of parent completing the questionnaire) and socioeconomic status (main occupation, education level, and income per month). The questionnaire for parents/guardians consisted of three parts. The first part collected demographic and socioeconomic information. The second part comprised 10 “yes/no” questions assessing knowledge about head lice, where incorrect answers were scored as zero, and correct answers were scored as one. The third part consisted of 10 “agree/disagree” questions assessing attitudes towards head lice, which were adapted from previously reported measures [32]. For purposes of data analysis, the knowledge level of parents was categorized as poor (scores less than or equal to 5), moderate (scores between 6 and 7), and high (scores between 8 and 10). The attitude towards head lice was assessed based on numerical data and percentages. School directors were asked in face-to-face discussions about the presence or absence of a school health policy specifically focusing on head louse screening and treatment, and the reasons behind their respective policies.

### 2.5. Statistical Analysis

In this study, the primary outcome was the prevalence of pediculosis, while the secondary outcomes included associated factors, parental knowledge and attitudes, and school policy status. The prevalence of pediculosis was calculated as the number of infested children divided by the total sample collection at the time point, multiplied by one hundred. Chi-square test was used to compare prevalence across groups. Demographic and socioeconomic data were presented using percentages, means, standard deviations (SD), and participant counts. The relationship between pediculosis and related factors was examined using logistic regression analysis. Variable factors with *p*-values ≤ 0.1 were initially selected using backward elimination. Given that participants were clustered within schools, a generalized linear mixed model (GLMM) with school included as a random effect was subsequently applied to account for potential non-independence of observations. Both approaches were compared to evaluate the robustness of findings. The analysis included eight schools (clusters), and although the number of clusters was relatively small, the mixed-effects model was used as an exploratory approach to account for potential clustering. The presence of clustering was assessed using a likelihood ratio (LR) test comparing the mixed-effects model with the logistic model and by estimating the intra-class correlation coefficient (ICC). Adjusted odds ratios (adjusted OR) with 95% confidence intervals (CI) were reported. Statistical significance was considered at *p* < 0.05. All analyses were performed using the STATA package version 10.1 (StataCorp LLC, College Station, TX, USA).

## 3. Results

### 3.1. Study Population and Characteristics

All eligible participants were girls from primary schools, and all provided informed consent to participate. Of the 510 eligible students, 494 were included in the analysis (96.9%), while 16 were excluded due to absence on the day of examination. Participants were distributed across Chonburi (*n* = 199), Nakhon Si Thammarat (*n* = 144), and Maha Sarakham (*n* = 151) Provinces.

The demographic characteristics of children (including hairstyle, class level, and parental marital status) and socioeconomic factors (parental occupation, parental education level, and income of parents/guardians) based on the combined data from all provinces are provided in Table 1. Additionally, Table S1 presents the same variables analyzed separately for each province. These data indicated statistically significant differences between provinces (*p* < 0.05). Socioeconomic factors (parental education level, occupation, and income) from three provinces were different.

### 3.2. Prevalence of Pediculosis in Schoolchildren

The overall prevalence of pediculosis was 50.81% (251/494; 95% CI: 46.31–55.20). Prevalence varied significantly across provinces (*p* < 0.001), with the highest rate observed in Nakhon Si Thammarat Province (69.44%; 100/144; 95% CI: 61.22–76.84), followed by Maha Sarakham Province (58.94%; 89/151; 95% CI: 50.65–66.87), and the lowest in Chonburi Province (31.16%; 62/199; 95% CI: 24.79–38.09) (Table 2).

### 3.3. Factors Associated with Pediculosis

Univariable analysis identified several factors associated with pediculosis, including class level, hair-washing practices, parental income, and history of herbal treatment use (Table S2). After accounting for clustering at the school level using a generalized linear mixed model (GLMM), only class level and hair-washing behavior remained significantly associated with pediculosis (Table 3). Compared with kindergarten children, those in elementary grades 1–3 had higher odds of infestation (adjusted OR = 3.09; 95% CI: 1.31–7.29; *p* = 0.010). Children who washed their hair by themselves were also more likely to be infested compared with those assisted by parents/guardians (adjusted OR = 2.93; 95% CI: 1.57–5.49; *p* = 0.001).

In contrast, the associations observed for parental income and herbal treatment use in the univariable analysis were attenuated and no longer statistically significant after accounting for clustering. A significant clustering effect at the school level was identified (likelihood ratio test *p* = 0.007), with an intra-class correlation coefficient (ICC) of 0.059 (95% CI: 0.008–0.332), indicating that part of the variability in pediculosis was attributable to differences between schools.

### 3.4. Parental Knowledge and Attitudes About Head Lice

The overall knowledge level of parents/guardians regarding various aspects of head lice, including biology, life cycle, mode of transmission, prevention, and treatment, was classed as moderate (Mean ± SD: 6.34 ±1.14). About 64.17% of parents/guardians were moderately knowledgeable, followed by poor (20.24%) and high (15.59%) levels of knowledge (Table S3). Common misconceptions included beliefs that head lice can jump and that they have only two life stages. A high proportion of parents believed that daily hair washing could prevent infestation (84.62%) and that herbal treatments were more effective than chemical products (80.97%) (Table 4). These findings reflect commonly held perceptions, although evidence supporting their effectiveness remains limited or inconclusive. The analysis conducted within each province yielded similar results to the combined analysis (Table S4).

### 3.5. School Health Policy

The survey responses from school directors regarding school health policies revealed that most schools did not have an existing policy to conduct screening and treatment for head lice on an annual basis.

## 4. Discussion

The findings of this study shed light on several important aspects of pediculosis among primary schoolgirls in Thailand, offering insights into its prevalence, associated factors, and implications for control measures. The study was conducted simultaneously in each school and employed a consistent duration time and diagnostic tools throughout. The overall prevalence of pediculosis in the present study was found to be 50.81%. Notably, the highest prevalence was observed in Nakhon Si Thammarat Province (69.44%) followed by Maha Sarakham Province (58.94%), while the lowest prevalence was found in Chonburi Province (31.16%). These findings revealed a statistically significant difference in the prevalence of pediculosis between locations within Thailand and aligned with previous studies conducted in various provinces of Thailand [32,33,34,35].

A higher prevalence of pediculosis was observed in rural areas of Nakhon Si Thammarat and Maha Sarakham Provinces compared with the urban area of Chonburi Province. A similar pattern was noted within Maha Sarakham Province [39], where a previous study conducted in an urban setting reported a lower prevalence (23.38%), whereas the present study identified a markedly higher prevalence in rural schools (58.94%). However, because urban and rural settings were not concurrently sampled within each province, the effects of geographic region and level of urbanization could not be disentangled. Therefore, comparisons across study areas should be interpreted as descriptive rather than indicative of causal relationships. Nevertheless, the observed pattern is consistent with previous studies from various countries reporting higher pediculosis prevalence in rural settings compared with urban areas [24,28,40], suggesting that differences in local context may contribute to variation in infestation rates. Variation was also observed within Chonburi Province. The prevalence reported in the present study (31.16%) was lower than that observed in a school attended by both Thai and migrant children (46.00%) [41], but higher than that reported in another urban setting (23.85%) [42]. These differences indicate that heterogeneity in social and living conditions, even within the same urban area, may influence infestation dynamics [43].

It is important to note that the prevalence of pediculosis across the sampled provinces in the present study remained high. Differences in prevalence may reflect contextual factors that were not directly measured in this study. Previous research has suggested that environmental conditions, including season and humidity, may influence pediculosis dynamics, for example, through effects on egg hatching and survival [44]. However, as this study was conducted between January and March under broadly similar warm and humid conditions across provinces, the role of seasonal or climatic factors could not be assessed. Therefore, these factors should be interpreted as potential contextual influences rather than confirmed determinants. Longitudinal studies conducted across different seasons and settings would be needed to better understand their contribution to pediculosis transmission.

In Thailand, it is common for children to reside with their grandparents, especially in rural areas. This arrangement typically occurs when parents migrate to find employment or due to problematic family relationships [45,46]. In this study, demographic factors (parents/guardians age groups, marital status, and gender of parents/guardians) and socioeconomic factors (parents/guardians education level, and occupation groups) were not significantly related to pediculosis whether analyzed separately or with all data combined. The relationship between head louse infestations and demographic factors of parents/guardians is likely weak because the primary determinants of pediculosis are related to the behavior of children, environmental conditions, and the overall approach to lice management. Other demographic characteristic factors of children, such as hair length, were also not found to be significant in this study. The results are similar to previous studies conducted in rural and suburban areas of Thailand [32,33,34]. In contrast, a previous case–control study in urban areas in Maha Sarakham Province, northeastern Thailand, found that hair length was related to head louse infestations among girl schoolchildren, and that long hair increased the risk of transmission from other people [39]. In the present study, however, hair length was not identified as a significant determinant after accounting for contextual factors. This discrepancy may reflect differences in study design, sampling approaches, and school-level practices, including grooming policies such as tying long hair. Importantly, both short and long hair can be infested when head-to-head contact occurs, suggesting that contact patterns may be more influential than hair length alone. Nevertheless, hair length may still play a role in facilitating detection and removal, as longer hair can make systematic inspection and combing more challenging. Therefore, its influence may be indirect and context-dependent rather than a consistent independent risk factor.

Children who washed their hair by themselves were more likely to be infested than those assisted by parents or caregivers. This may reflect less thorough washing or reduced supervision. Although school-aged children (grades 1–6) often perform personal hygiene independently, the effectiveness of these practices may vary. In contrast, younger children may benefit from closer supervision, which could contribute to the lower prevalence observed in this group. In province-specific analyses, no statistically significant difference in pediculosis prevalence was observed between self-performed and caregiver-assisted hair washing in Nakhon Si Thammarat Province. This is consistent with findings from Chiang Rai Province, northern Thailand [33], where high infestation rates were reported regardless of who performed hair washing. This might be attributed to parents/guardians in these settings not being meticulous in the hair-washing process, or other factors may contribute to re-infestations. Additionally, head louse infestations are often asymptomatic or present mild symptoms (itching), and children may not communicate this to their parents/guardians, contributing to the sustained prevalence of pediculosis in children [13].

This study also revealed gaps in parental knowledge and attitude regarding head lice. Despite parents/guardians having a moderate level of knowledge, misunderstandings about head lice biology, transmission, and treatment were prevalent. A large proportion of parents reported beliefs related to prevention and treatment, including the perception that daily hair washing may prevent infestation (84.62%) and that herbal treatments are more effective than chemical products (80.97%). These findings reflect commonly held practices and perceptions, although the evidence supporting their effectiveness remains limited or inconclusive. There is currently no clear evidence to support whether regular hair washing can effectively remove head lice or how frequently it should be performed. Generally, washing hair with shampoo is a quick process, and head lice can survive in water for several hours [14,15]. Many pediculicide products have limited effectiveness in killing louse eggs and removing them from the hair shaft [12]. Genetic resistance to pyrethroid, a commonly used insecticide class, has been reported in head lice in Thailand [18,47], and the inefficacy of some pediculicide-based herbal shampoo products, available in the market, to kill head lice has also been reported [29,30,42]. These factors may contribute to the persistence of head lice on children, even with regular hair washing. Therefore, providing accurate and updated information about head louse treatment to parents/guardians, along with ensuring the availability of effective and safe pediculicide products, is imperative.

While several individual-level factors, including parental income and the use of herbal treatments, appeared to be associated with pediculosis in the univariable analyses, these associations should be interpreted with caution. When accounting for clustering at the school level, the magnitude of these associations was attenuated, suggesting that they may partly reflect contextual differences between schools rather than independent individual-level determinants. The presence of a statistically significant school-level effect, despite a modest magnitude (ICC = 0.059), indicates that pediculosis transmission may be influenced not only by individual behaviors but also by shared environmental and institutional factors within schools. These may include hygiene practices, patterns of close contact among children, and school-level approaches to screening and management. These findings reinforce the notion that interventions focusing solely on individual behaviors may be insufficient. Instead, coordinated strategies implemented at the school level may be required to interrupt transmission more effectively within school settings.

The school health policy concerning screening and treatment for head lice has a crucial role in controlling re-infestation and distribution among schoolchildren. A high prevalence of pediculosis at school might indirectly affect the control of head lice. All schools that we surveyed lacked a policy to check for and treat head lice each year. Additionally, about 92.91% of parents/guardians believed that head lice can be transmitted from other children at school. However, children could also become infested with head lice at home, where transmission could still occur [48]. Physical contact between infested children from different schools in the community could also lead to the transfer of head lice. These findings suggest that public health authorities may consider implementing routine screening policies and coordinated management approaches across schools, households, and communities.

### 4.1. Implications for Effective Control

The results highlight several implications for effective control of pediculosis, suggesting that improvements may be achieved through strengthening caregiver education, promoting consistent treatment practices with effective products, and enhancing systematic school-based management approaches. Controlling head louse infestations and re-infestations should combine health education and provision of effective pediculicide products. Although health education could improve knowledge and attitudes in children [37], failure to use an effective product for the treatment of head lice would fail to reduce most of the infestation. Education should therefore focus on correct treatment practices, including appropriate product use, adherence to recommended regimens, and avoidance of repeated misuse [13]. In Thailand, where reduced efficacy of commonly used pyrethroid-based pediculicides and herbal-based shampoo has been reported [29], treatments with different mechanisms of action, particularly ivermectin, abametapir, and dimeticone-based formulations, may warrant consideration, although their availability remains limited. In the interim, herbal products should be used only if they have been rigorously evaluated and demonstrated to be effective and should be implemented alongside coordinated treatment approaches. Given the observed association with self-performed hygiene, promoting feasible and age-appropriate self-care practices may represent a relevant component of control strategies. Although children are generally expected to perform basic personal hygiene independently, the effectiveness of these practices may vary. The present findings suggest that inconsistent or suboptimal self-care may contribute to infestation risk. Therefore, if highly effective and practical treatment methods that can be reliably performed by children are available, they may substantially enhance control efforts. At the same time, parental involvement remains important, particularly in ensuring thorough treatment application and adherence. Providing parents and caregivers with accurate, up-to-date information on head lice biology, transmission, and treatment may help address misunderstandings and improve management practices. Beyond individual- and household-level measures, the findings highlight the importance of coordinated approaches at the school and community levels. As head lice transmission occurs within interconnected social networks, interventions targeting individuals alone may be insufficient to interrupt ongoing transmission. Synchronized strategies, such as implementing a predefined “lice week,” aim to coordinate treatment across households within a defined time frame, thereby reducing the likelihood of reinfestation. Evidence from previous studies and modeling suggests that such approaches can improve control outcomes by disrupting transmission cycles [3,11,13]. In the present study, the observed clustering at the school level supports the relevance of these coordinated strategies, indicating that transmission may be influenced not only by individual behaviors but also by shared environments and contact patterns within schools. These findings suggest that effective control may benefit from combining individual-level practices with structured, school-based or community-wide interventions tailored to local contexts.

### 4.2. Limitations and Future Research

This study has several limitations. First, as a cross-sectional design conducted between January and March 2023, it captures observations at a single point in time and therefore cannot establish causal relationships between potential determinants and pediculosis; only associations can be inferred. Second, although clustering at the school level was accounted for using a mixed-effects model, the relatively small number of clusters may limit the precision of school-level estimates, and residual confounding at both individual and contextual levels cannot be entirely excluded. Third, the study focused exclusively on primary schoolgirls, which may limit the generalizability of the findings to boys and other age groups. However, this approach was justified by the higher burden of pediculosis among girls in the study setting. Fourth, information on hygiene practices and parental beliefs was self-reported and may be subject to recall or social desirability bias. Fifth, data on household-level factors, including infestation among family members, were not collected, as the study was designed to focus on school-based transmission. Finally, the study did not include both rural and urban schools within each province, and northern regions were not represented. Data were collected from only three of Thailand’s 77 provinces, representing three geographic regions of the country. These factors may limit the representativeness of the findings at the national level. Future research should address these limitations by adopting longitudinal or repeated survey designs to capture temporal dynamics and strengthen causal interpretation. Expanding the study population to include both boy and girl students, a broader age range, and household members would improve generalizability and allow assessment of household transmission. More balanced sampling of rural and urban schools within each province and across all geographic regions, including northern Thailand, together with formal assessment of inter-rater reliability, would enhance the robustness and national relevance of future findings.

## 5. Conclusions

The overall prevalence of pediculosis was high across the sampled provinces. After accounting for clustering at the school level, only class level and self-performed hair washing were consistently associated with infestation, while other factors were not retained. Parental knowledge was moderate, and beliefs regarding prevention and treatment varied, with limited supporting evidence. None of the participating schools had routine head lice screening policies. These findings suggest that control efforts should not rely on individual practices alone. School-level approaches, including routine screening and coordinated management, together with improved access to effective treatment and targeted health education, may help reduce the burden of pediculosis in this setting.

## Figures and Tables

**Table 1:** Demographic and socioeconomic characteristics among girl schoolchildren in this study (*n* = 494).

Variables	*n*	Percentage
Student class levels		
Kindergarten 1–3	64	12.96
Elementary grade 1–3	176	35.63
Elementary grade 4–6	254	51.41
Religion		
Buddhism	369	74.70
Islam	125	25.30
Length of hair		
Short (Equal shoulders)	334	67.61
Long	160	32.39
Gender of parent/guardian who responded to questionnaire		
Male	46	9.31
Female	448	90.69
Age of parents/guardians (years)		
<30	47	9.51
30–39	193	39.07
40–49	168	34.01
50–59	55	11.13
≥60	31	6.28
Mean ± S.D. (40.77 ± 9.92)		
Status of parents/guardians		
Married	403	81.58
Divorce	32	6.48
Other	59	11.94
Parent’s level of education		
Illiterate	8	1.62
Elementary	31	6.28
Secondary 3	192	38.87
Secondary 6	128	25.91
Higher than secondary	135	27.33
Parent’s occupation		
Government official	14	2.84
Agricultural	57	11.56
Retail business	122	24.75
Worker	225	45.64
Other	75	15.21
Income per month (Baht) of the parent completing the questionnaire		
≥10,000	165	33.40
<10,000	329	66.60

**Table 2:** Prevalence of pediculosis among girl schoolchildren from different geographic areas of Thailand.

Provinces	*n*	Pediculosis		*X* ^2^	***p***-Value ^a^
		Yes (%)	No (%)		
Chonburi	199	62 (31.16)	137 (68.84)	54.75	<0.001
Maha Sarakham	151	89 (58.94)	62 (41.06)		
Nakhon Si Thammarat	144	100 (69.44)	44 (30.56)		
All	494	251(50.81)	243 (49.19)		

^a^ Based on the Chi-square test.

**Table 3:** Generalized linear mixed model analysis of factors associated with pediculosis among schoolchildren (*n* = 494).

Variables	Pediculosis		Crude OR ^a^	Adjusted OR ^b^	95% CI	*p*-Value
	Yes (%)	No (%)				
Student class levels						
Kindergarten 1–3	10 (15.63)	54 (84.38)	1			
Elementary grade 1–3	111 (63.07)	65 (36.93)	9.22	3.09	1.31, 7.29	0.010
Elementary grade 4–6	130 (51.18)	124 (48.82)	5.66	1.22	0.47, 3.16	0.679
Personal hair washing						
By parents/guardians	34 (29.82)	80 (70.18)	1			
By themselves	217 (57.11)	163 (42.89)	3.13	2.93	1.57, 5.49	0.001
History of pediculicide use						
No	107 (42.80)	143 (57.20)	1			
Yes, by chemical	45 (44.55)	56 (55.45)	1.07	0.49	0.67, 1.94	0.874
Yes, by herb	99 (69.23)	44 (30.77)	3.01	1.89	0.98, 2.81	0.059
Parental income per month (baht)						
≥10,000	62 (37.58)	103 (62.42)	1			
<10,000	189 (57.45)	140 (42.55)	2.24	1.07	0.64, 1.79	0.807

^a^ Crude odds ratios were estimated using univariable logistic regression analysis without accounting for clustering. ^b^ Adjusted odds ratios were obtained from a generalized linear mixed model (GLMM) with school included as a random effect to account for clustering of participants within schools. *p*-values were derived from the mixed-effects model. Statistical significance was set at *p* < 0.05.

**Table 4:** Attitudes about head lice among parents/guardians (*n* = 494).

Variables	Agree (%)	Disagree (%)
Head lice are nasty insects.	466 (94.33)	31 (6.27)
Head lice can cause anemia.	347 (70.24)	147 (29.76)
Head lice can make children stupid.	227 (45.95)	267 (54.05)
People infested with head lice are dirty.	372 (75.30)	122 (24.70)
Head lice should not be treated.	86 (17.41)	408 (82.59)
Pediculicidal compounds can kill all stages of head lice.	262 (53.04)	232 (46.96)
Head lice can be transmitted from other children at school.	459 (92.91)	35 (7.09)
Head lice can be acquired by sharing personal items.	444 (89.88)	50 (10.12)
Hair washing every day can prevent head louse infestation.	418 (84.62)	76 (15.38)
Herbal shampoo is more effective for killing head lice than are chemical pediculicide products.	400 (80.97)	94 (19.03)

## Data Availability

The original contributions presented in this study are included in the article/Supplementary Materials. Further inquiries can be directed to the corresponding author.

## Supplementary Materials

The following supporting information can be downloaded at https://www.mdpi.com/article/10.3390/insects17040413/s1, File S1: Flow diagram of multi-stage sampling and participant recruitment; Table S1: Data concerning pediculosis in girl schoolchildren from each province surveyed, and combined data of three provinces; Table S2: Univariate logistic regression analyses of the association between pediculosis and demographic characteristics and socioeconomic factors of children (*n* = 494); Table S3: Knowledge levels of parents/guardians about head lice (*n* = 494); Table S4. Attitudes about head lice of parents/guardians (*n* = 494).
