# Supplementary material for: Epidemiology, Associated Factors and Implications for Effective Control of Pediculosis Among Primary Schoolgirls in Thailand: A Cross-Sectional Study
Source: Insects. 2026 Apr 10;17(4):413. doi: 10.3390/insects17040413 (PMC13116654; doi:10.3390/insects17040413)
Supplement: Supplementary file 1 [file insects-17-00413-s001.zip › Table S1 Yingklang.pdf]

Table S1 Data concerning pediculosis in girl schoolchildren from each province surveyed, and combined data of three provinces.

Variables	All (n= 494)	Pediculosis (All)	*p*-value[a]	Total (n = 199)	Chonburi Province	*p*-value[a]	Total (n =151)	Maha Sarakham Province	*p*-value[a]	Total (n =144)	Nakhon Si Thammarat Province	*p*-value[a]
		Infested n (%)			Infested n (%)			Infested n (%)			Infested n (%)	
**Student class level**			**<0.001**			**<0.001**			**0.007**			0.705
Kindergarten 1-3	64	10 (15.63)		55	8 (14.55)#		9	2 (22.22)		-	-	
Elementary grade 1-3	176	111 (63.07)		65	31 (47.69)		52	38 (73.08)		59	42 (71.19)	
Elementary grade 4-6	254	130 (51.18)		79	23 (29.11)		90	49 (54.44)		85	58 (68.24)	
**Length of hair**			0.409			0.363			0.779			**0.031**
Short (not below shoulders)	334	174 (69.32)		149	49 (32.89)		71	41 (57.75)		114	84 (73.68)	
Long	160	77 (30.68)		50	13 (26.00)		80	48 (60.00)		30	16 (53.33)	
**Gender of parents/guardians**			0.080			0.378			0.988			0.348
Male	46	29 (63.04)		9	4 (44.44)		22	13 (59.09)		15	12 (80.00)	
Female	448	222 (49.55)		190	58 (30.53)		129	76 (58.91)		129	88 (68.22)	
**Age of parents/guardians (years)**			0.096			0.147			0.788			0.636
≤30	47	16 (34.04)		31	6 (19.35)		6	4 (66.67)		10	6 (60.00)	
31- 39	193	100 (51.81)		89	31 (34.83)		48	31 (64.58)		56	38 (67.86)	
40-49	168	87 (51.79)		62	23 (37.10)		55	30 (54.55)		51	34 (66.67)	
50-59	55	28 (50.91)		12	1 (8.33)		28	15 (53.57)		15	12 (80.00)	
≥60	31	20 (64.52)		5	1 (20.00)		14	9 (64.29)		12	10 (83.33)	

**Status of parents/guardians**			0.518			0.527			0.530			0.087
Married	403	206 (51.12)		169	54 (31.95)		120	69 (57.50)		114	83 (72.81)	
Divorce	32	15 (46.88)		7	3 (42.86)		10	5 (50.00)		15	7 (46.67)	
Other	59	30 (49.41)		23	5 (21.74)		21	15 (71.43)		15	10 (66.67)	
**Education level of parents/guardians**			0.599			0.352			0.787			0.003
Illiterate	8	2 (0.80)		8	2 (25.00)		-	-		-	-	
Elementary	31	15 (5.98)		27	12 (44.44)		1	1 (100)		3	2 (66.67)	
Secondary 3	192	102 (40.64)		71	17 (23.94)		55	32 (58.18)		66	53 (80.30)	
Secondary 6	128	64 (25.50)		73	25 (34.25)		30	19 (63.33)		25	20 (80.00)	
Higher than secondary	135	68 (27.09)		20	6 (31.16)		65	37 (56.92)		50	25 (50.00)	
**Occupation of parents/guardians**			0.602			0.749			0.890			0.537
Government official	14	8 (57.14)		4	1 (25.00)		4	3 (75.00)		6	4 (66.67)	
Agricultural	57	33 (57.89)		-	-		54	30 (55.56)		3	3 (100.00)	
Retail business	122	56 (45.90)		53	14 (26.42)		29	17 (58.62)		40	25 (62.50)	
Worker	225	117 (52.00)		104	33 (31.73)		51	32 (62.75)		70	52 (74.29)	
Other	75	37 (49.33)		38	14 (36.84)		13	7 (53.85)		24	16 (66.67)	
**Parent’s income per month (baht)**			**<0.001**			0.715			0.756			0.348
≥ 10,000	165	62 (37.58)		132	40 (30.30)		18	10 (55.56)		15	12 (80.00)	
< 10,000	329	189 (57.45)		67	22 (32.84)		133	79 (59.40)		129	88 (68.22)	
**History of using pediculicides**			**<0.001**			0.474			0.738			0.119
No	250	107 (42.80)		126	37 (29.37)		82	46 (56.10)		42	24 (57.14)	
Yes, by chemical	101	45 (44.55)		73	25 (34.25)		11	7 (63.64)		17	13 (76.47)	
Yes, by herb	143	99 (69.23)		-	-		58	36 (62.07)		85	63 (77.12)	
**Personal hair washing**			**<0.001**			**0.016**			**0.001**			0.330
By parents/guardians	114	34 (29.82)		69	14 (20.29)		22	6 (27.27)		23	14 (60.87)	

By themselves	380	217 (57.11)		130	48 (36.92)		129	83 (64.34)		121	86 (71.07)	

[a] *Based on the Chi-square goodness of fit test.*
