# Supplementary material for: Epidemiology, Associated Factors and Implications for Effective Control of Pediculosis Among Primary Schoolgirls in Thailand: A Cross-Sectional Study
Source: Insects. 2026 Apr 10;17(4):413. doi: 10.3390/insects17040413 (PMC13116654; doi:10.3390/insects17040413)
Supplement: Supplementary file 1 [file insects-17-00413-s001.zip › Table S2 Yingklang.pdf]

Table S2 Univariate logistic regression analyses of the association between pediculosis and demographic characteristics and socioeconomic factors of children (n= 494).

Variables	Total	Pediculosis		Crude OR	95%CI	*p*-value
		Yes (%)	No (%)			
**Student class level**						
Kindergarten 1-3	64	10 (15.63)	54 (84.38)	1		**<0.001**
Elementary grade 1-3	176	111 (63.07)	65 (36.93)	9.22	4.40, 19.35	
Elementary grade 4-6	254	130 (51.18)	124 (48.82)	5.66	2.76, 11.61	
**Length of hair**						
Short (not below shoulders)	334	174 (69.32)	160 (65.84)	1		0.409
Long	160	77 (30.68)	83 (34.16)	0.85	0.59, 1.24	
**Gender of parents/guardians responding to questionnaire**						
Male	46	29 (63.04)	17 (36.96)	1		0.080
Female	448	222 (49.55)	226 (50.45)	0.58	0.31, 1.08	
**Age of parents/guardians (years)**						
≤30	47	16 (34.04)	31 (65.96)	1		0.096
31- 39	193	100 (51.81)	93 (48.19)	2.08	1.07, 4.06	
40-49	168	87 (51.79)	81 (48.21)	2.08	1.06, 4.09	
50-59	55	28 (50.91)	27 (49.09)	2.01	0.90, 4.48	
≥60	31	20 (64.52)	11 (35.48)	3.52	1.36, 9.12	
**Status of parents/guardians**						
Married	403	206 (51.12)	197 (48.88)	1		0.518
Divorce	32	15 (46.88)	17 (53.13)	0.84	0.41, 1.74	
Other	59	30 (49.41)	29 (50.59)	0.63	0.28, 1.45	
**Education level of parents/guardians**						
Illiterate	8	2 (0.80)	6 (2.47)	1		0.599
Elementary	31	15 (5.98)	16 (6.58)	2.81	0.49, 16.16	
Secondary 3	192	102 (40.64)	90 (37.04)	3.40	0.67, 17.27	
Secondary 6	128	64 (25.50)	64 (26.34)	3.00	0.58, 15.43	
Higher than secondary	135	68 (27.09)	67 (27.57)	3.04	0.59, 15.63	
**Occupation of parents/guardians**						

Government official	14	8 (57.14)	6 (42.86)	1		0.602
Agricultural	57	33 (57.89)	24 (42.11)	1.03	0.32, 3.36	
Retail business	122	56 (45.90)	66 (54.10)	0.64	0.21, 1.94	
Worker	225	117 (52.00)	108 (48.00)	0.81	0.27, 2.41	
Other	75	37 (49.33)	38 (50.67)	0.73	0.23, 2.31	
**Parent's income per month (baht)**						
≥ 10,000	165	62 (37.58)	103 (62.42)	1		**<0.001**
< 10,000	329	189 (57.45)	140 (42.55)	2.24	1.53, 3.29	
**History of using pediculicides**						
No	250	107 (42.80)	143 (57.20)	1		**<0.001**
Yes, by chemical	101	45 (44.55)	56 (55.45)	1.07	0.67, 1.71	
Yes, by herb	143	99 (69.23)	44 (30.77)	3.01	1.95, 4.64	
**Personal hair washing**						
By parents/guardians	114	34 (29.82)	80 (70.18)	1		**<0.001**
By themselves	380	217 (57.11)	163 (42.89)	3.13	1.99, 4.91	

#
