# Supplementary material for: Epidemiology, Associated Factors and Implications for Effective Control of Pediculosis Among Primary Schoolgirls in Thailand: A Cross-Sectional Study
Source: Insects. 2026 Apr 10;17(4):413. doi: 10.3390/insects17040413 (PMC13116654; doi:10.3390/insects17040413)
Supplement: Supplementary file 1 [file insects-17-00413-s001.zip › Table S3 Yingklang.pdf]

Table S3 Knowledge levels of parents/guardians about head lice (n = 494)

**Questions**	**Incorrect answer n (%)**	**Correct answer n (%)**
1. Head lice can jump.	264 (53.44)	230 (46.56)
2. Head lice feed on human blood.	4 (0.81)	490 (99.19)
3. Head lice have only two stages (egg and adult).	270 (54.66)	224 (45.34)
4. Viable lice egg have white or yellow color.	18 (3.64)	476 (96.36)
5. Head lice can spread from pets or farm animals.	258 (52.23)	236 (47.77)
6. Treatment with pediculicides must be done twice.	376 (76.11)	118 (23.89)
7. Head lice can spread through direct head-to-head contact with infested people.	15 (3.04)	479 (96.96)
8. Wet combing is the best method for identifying head lice.	437 (88.46)	57 (11.54)
9. Some available pediculicides kill all lice eggs.	248 (50.20)	246 (49.80)
10. Head lice will survive in water for several hours.	253 (51.21)	241 (48.79)

**Knowledge levels**	**n**	**Percentage**
High level (score 8-10)	77	15.59
Moderate level (score 6-7)	317	64.17
Poor level (score 0-5)	100	20.24
**Mean± SD.: 6.34± 1.14**		
