# Supplementary material for: Epidemiology, Associated Factors and Implications for Effective Control of Pediculosis Among Primary Schoolgirls in Thailand: A Cross-Sectional Study
Source: Insects. 2026 Apr 10;17(4):413. doi: 10.3390/insects17040413 (PMC13116654; doi:10.3390/insects17040413)
Supplement: Supplementary file 1 [file insects-17-00413-s001.zip › Table S4 Yingklang.pdf]

Table S4 Attitudes about head lice of parents/guardians (n = 494).

Variables	Chonburi (n=199)		Maha Sarakham (n=151)		Nakhon Si Thammarat (n=144)		All	
	Agree n (%)	Disagree n (%)	Agree n (%)	Disagree n (%)	Agree n (%)	Disagree n (%)	Agree n (%)	Disagree n (%)
1. Head lice are nasty insects								
	175 (87.94)	24 (12.06)	147 (97.35)	4 (2.65)	144 (97.92)	3 (2.08)	466 (94.33)	31 (6.27)
2. Head lice can make children anemia.								
	141 (70.85)	58 (29.15)	102 (67.55)	49 (32.45)	104 (72.22)	40 (27.78)	347 (70.24)	147 (29.76)
3. Head lice can make children stupid.								
	81 (40.70)	118 (59.30)	82 (54.30)	69 (45.70)	64 (44.44)	80 (55.56)	227 (45.95)	267 (54.05)
4. People infested with head lice are dirty.								
	150 (75.38)	49 (24.62)	121 (80.13)	30 (19.87)	101 (70.14)	43 (29.86)	372 (75.30)	122 (24.70)
5. Head lice should not be treated.								
	48 (24.12)	151 (75.88)	26 (17.22)	125 (82.78)	12 (8.33)	132 (91.67)	86 (17.41)	408 (82.59)
6. Pediculicidal compounds can kill all stages of head lice.								
	117 (58.79)	82 (41.21)	76 (50.33)	75 (49.67)	69 (47.92)	75 (52.08)	262 (53.04)	232 (46.96)
7. Head lice can be transmitted from other children at school.								
	189 (94.97)	10 (5.03)	140 (92.72)	11 (7.28)	130 (90.28)	14 (9.27)	459 (92.91)	35 (7.09)
8. Head lice can be acquired by sharing personal items.								
	183 (91.96)	16 (8.04)	132 (87.42)	19 (12.58)	129 (89.58)	15 (10.42)	444 (89.88)	50 (10.12)
9. Hair washing every day can prevent head-louse infestations?								
	166 (83.42)	33 (16.58)	136 (90.07)	15 (9.93)	116 (80.56)	28 (19.44)	418 (84.62)	76 (15.38)
10. Herbal shampoo is more effective at killing head lice than are chemical pediculicide products.								
	150 (75.38)	49 (24.62)	133 (88.08)	18 (11.92)	117 (81.25)	27 (18.75)	400 (80.97)	94 (19.03)
