# Supplementary figures and images for: Epidemiology, Associated Factors and Implications for Effective Control of Pediculosis Among Primary Schoolgirls in Thailand: A Cross-Sectional Study
Source: Insects. 2026 Apr 10;17(4):413. doi: 10.3390/insects17040413 (PMC13116654; doi:10.3390/insects17040413)

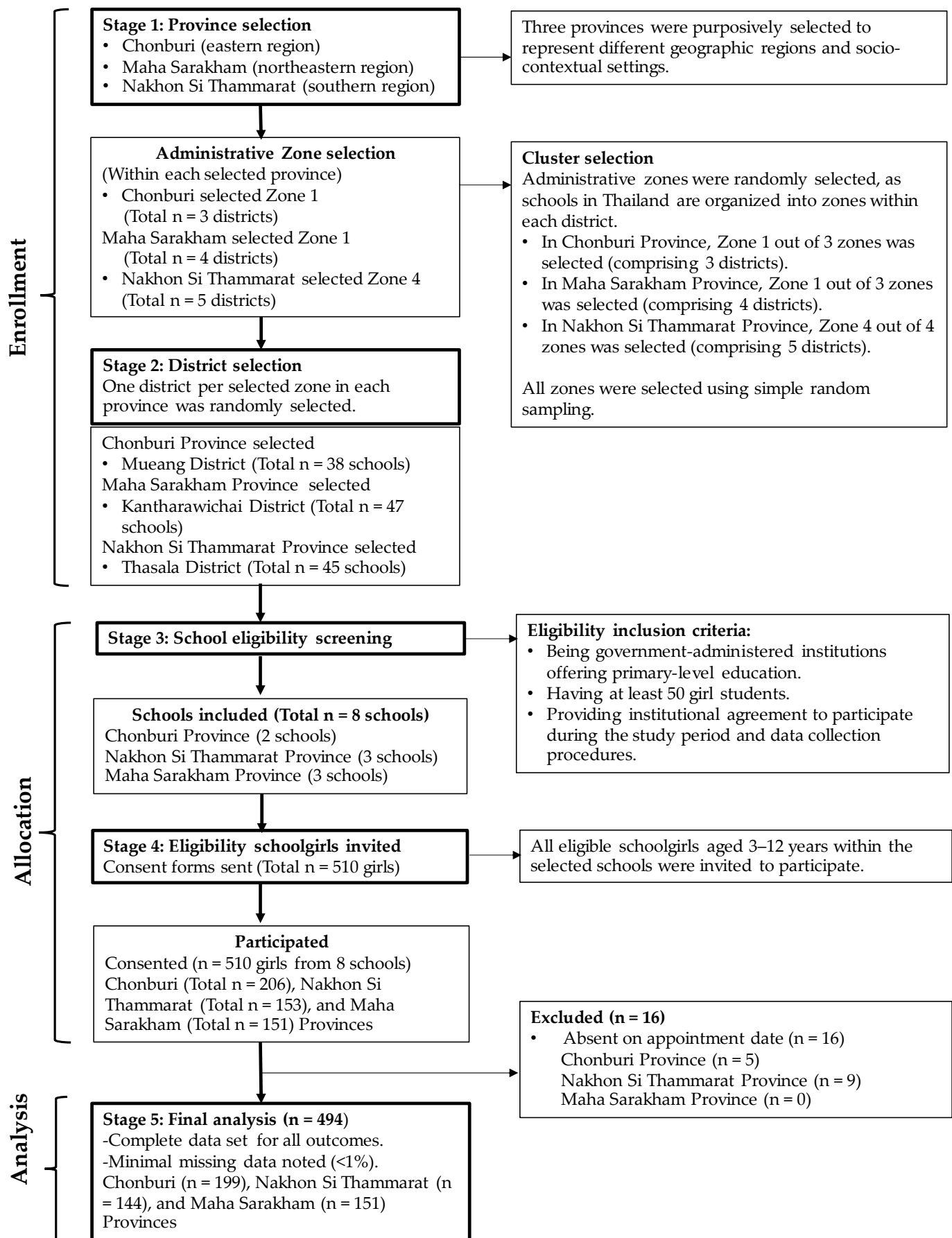

**Flow diagram of multi-stage sampling and participant recruitment.**

Supplement: Supplementary file 1 [file insects-17-00413-s001.zip › File S1.pdf]
